# CNVoyant: A Highly Performant and Explainable Multi-Classifier Machine Learning Approach for Determining the Clinical Significance of Copy Number Variants

**DOI:** 10.21203/rs.3.rs-4308324/v1

**Published:** 2024-04-30

**Authors:** Robert J. Schuetz, Defne Ceyhan, Austin A. Antoniou, Bimal P. Chaudhari, Peter White

**Affiliations:** The Abigail Wexner Research Institute at Nationwide Children’s Hospital; The Abigail Wexner Research Institute at Nationwide Children’s Hospital; The Abigail Wexner Research Institute at Nationwide Children’s Hospital; The Abigail Wexner Research Institute at Nationwide Children’s Hospital; The Abigail Wexner Research Institute at Nationwide Children’s Hospital

## Abstract

The precise classification of copy number variants (**CNVs**) presents a significant challenge in genomic medicine, primarily due to the complex nature of CNVs and their diverse impact on genetic disorders. This complexity is compounded by the limitations of existing methods in accurately distinguishing between benign, uncertain, and pathogenic CNVs. Addressing this gap, we introduce CNVoyant, a machine learning-based multi-class framework designed to enhance the clinical significance classification of CNVs. Trained on a comprehensive dataset of 52,176 ClinVar entries across pathogenic, uncertain, and benign classifications, CNVoyant incorporates a broad spectrum of genomic features, including genome position, disease-gene annotations, dosage sensitivity, and conservation scores. Models to predict the clinical significance of copy number gains and losses were trained independently. Final models were selected after testing 29 machine learning architectures and 10,000 hyperparameter combinations each for deletions and duplications via 5-fold cross-validation. We validate the performance of the CNVoyant by leveraging a comprehensive set of 21,574 CNVs from the DECIPHER database, a highly regarded resource known for its extensive catalog of chromosomal imbalances linked to clinical outcomes. Compared to alternative approaches, CNVoyant shows marked improvements in precision-recall and ROC AUC metrics for binary pathogenic classifications while going one step further, offering multi-classification of clinical significance and corresponding SHAP explainability plots. This large-scale validation demonstrates CNVoyant’s superior accuracy and underscores its potential to aid genomic researchers and clinical geneticists in interpreting the clinical implications of real CNVs.

## Introduction

The establishment of reference genomes, sequencing technologies, and post-processing algorithms has ushered in an era where genetic variation is reliably detectable. Databases are maintained to define functional regions of the genome (O’Leary et al. 2016; Howe et al. 2021), catalog observed genetic variants (Sherry 2001; Exome Aggregation Consortium et al. 2016), record variant frequencies in different human populations (1000 Genomes Project Consortium et al. 2015; The UK10K Consortium et al. 2015; Koch 2020), and provide annotations regarding clinical significance (Landrum et al. 2018). However, entries in these resources favor smaller genetic changes, specifically single nucleotide variations (**SNVs**) and short insertions and deletions (**indels**). To date, these short variants have been the focus for clinical germline diagnoses in rare genetic diseases (**RGDs**); however, this may be a symptom of the limited capacity to discern the clinical significance of larger structural variants (**SV**s).

SVs cover larger segments of DNA and include, but are not limited to, copy number variants (**CNVs**), translocations, and inversions, all of which span at least 50 base pairs (**bp**) (1000 Genomes Project et al. 2011; MacDonald et al. 2014; Coutelier et al. 2022). The recent clinical adoption of genome sequencing (**GS**) has led to more reliable identification of SVs, at a much finer resolution than was possible with microarray technology (Sanchis-Juan et al. 2018; Gross et al. 2019; Liu et al. 2022). In contrast to exome sequencing (**ES**), which focuses on coding sequences, GS extends the breadth of detection to intronic and intergenic regions. This attribute is crucial given that SVs frequently occur in non-coding regions and can encompass multiple genes. Despite the newly available data, understanding the clinical significance of detected SVs remains a challenge. Compared to recurrent SVs with well-defined breakpoints, rare SVs are particularly difficult to interpret. Even when rare SVs are observed in population frequency databases, they are often not annotated for clinical significance (NHGRI Centers for Common Disease Genomics et al. 2020).

Despite the advent of next-generation sequencing (**NGS**), diagnostic genetic variants are typically only identified in 25–45% of patients undergoing GS for suspicion of having an RGD (Yang et al. 2014; Clark et al. 2018; Tan et al. 2019; McLean et al. 2023; Kumar et al. 2023). Reanalysis of undiagnosed cases has yielded additional diagnoses after considering CNVs, confirming reports of CNV involvement in RGDs (Hegele 2007; Weischenfeldt et al. 2013; Bergant et al. 2018). Recent efforts have been made to standardize the interpretation of CNVs, culminating in the American College of Medical Genetics and Genomics (**ACMG**) technical standards for interpreting CNVs (Riggs et al. 2020). These guidelines consider population frequency, the impact of overlapping functional regions, and previous clinical interpretation (Collins et al. 2020). Within these guidelines, these features are evaluated to determine haploinsufficiency **(HI)** and triplosensitivity (**TS**), the tolerance to regional losses or gains in the genome, respectively. Both concepts fall under the broader category of dosage sensitivity.

The ACMG guidelines, while highly valued in the clinical setting, tend to classify CNVs as having uncertain pathogenic significance (**VUS**), as observed in the algorithmic implementation of the guidelines, ClassifyCNV (Gurbich and Ilinsky 2020). In the interpretation setting, this limited specificity results in lengthy candidate CNV sets requiring review, with many benign CNVs being classified as VUS. To address this problem, several machine learning (**ML**) approaches have been proposed to enhance the precision of classifying the clinical significance of CNVs. These algorithms statistically learn from data elements related to dosage sensitivity, overlapping genes, population frequencies, regulatory elements, topologically associated domains, and genomic position to predict pathogenic potential (Zhang et al. 2021; Gažiová et al. 2022; Sharo et al. 2022; Hertzberg et al. 2022; Lv et al. 2023). None of these algorithms combine all informative features in a single model. Moreover, as is common for ML-based classifiers, they fail to provide prediction explanations for better interpretability as to why the algorithm chose the given classification for a CNV. Together, these two limitations motivated the development of an improved ML approach.

Here we introduce CNVoyant, a tree-based, multi-class clinical significance classifier that combines previously reported features with novel features to classify CNVs more accurately than previously published methods. CNVoyant provides prediction explanations and enhances the accuracy and granularity of clinical significance classifications, enabling rapid identification and interpretation of potentially pathogenic CNVs.

## Methods

**Figure 1:**
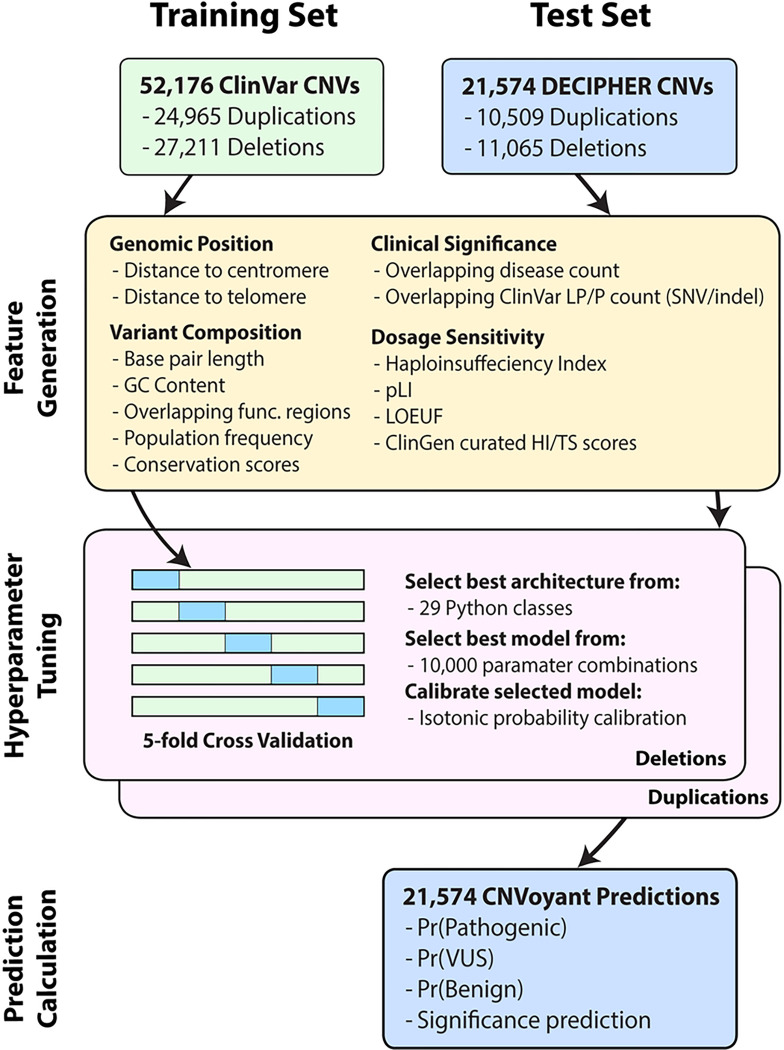
CNVoyant Development Framework The final CNVoyant models are a result of the illustrated machine learning pipeline and are designed to predict the pathogenicity of copy number variations (CNVs). The training set is comprised of 52,176 CNVs (24,965 duplications, 27,211 deletions) parsed from the Jan 2023 version of ClinVar, and the test set is comprised of 21,574 CNVs (10,509 duplications, 11,065 deletions) from DECIPHER v11.18. Features are generated from annotations related to genomic position, variant composition, clinical significance, and dosage sensitivity. Two models were trained to classify deletion and duplication events independently. Training data for each CNV type was partitioned into 5 cross folds. Accuracy metrics observed in each fold were utilized to (1) select the optimal architecture from 29 candidates, (2) select an optimal set of hyperparameters from 10,000 permutations, and (3) calibrate outputted probabilities to class distributions in the training data. The resulting models were used to generate probabilities of benign significance (Pr (Benign)), VUS (Pr (VUS)), and pathogenic significance (Pr (Pathogenic)) for CNVs in the test set. A clinical significance prediction is also provided by taking a maximum over the set of benign, VUS, and pathogenic probabilities. The CNVoyant output generated from the test set was later used for benchmarking.

The capacity of CNVoyant to classify the clinical significance of CNVs was tested in 21,574 CNVs curated from DECIPHER after training on 52,176 CNVs published in ClinVar (Fig. 1). This approach is consistent with previously reported comparisons of pathogenicity, where a set of CNVs are examined in a general context rather than focusing on individual patients. This also aligns with the previously cited ACMG technical guidelines, that recommend uncoupling CNV pathogenicity classification from the implications for a specific patient (Riggs et al. 2020). Features were generated to capture information related to genomic position, variant composition, overlapping functional annotation, population frequency, conservation, and dosage sensitivity.

### Training Dataset Curation

**Figure 2:**
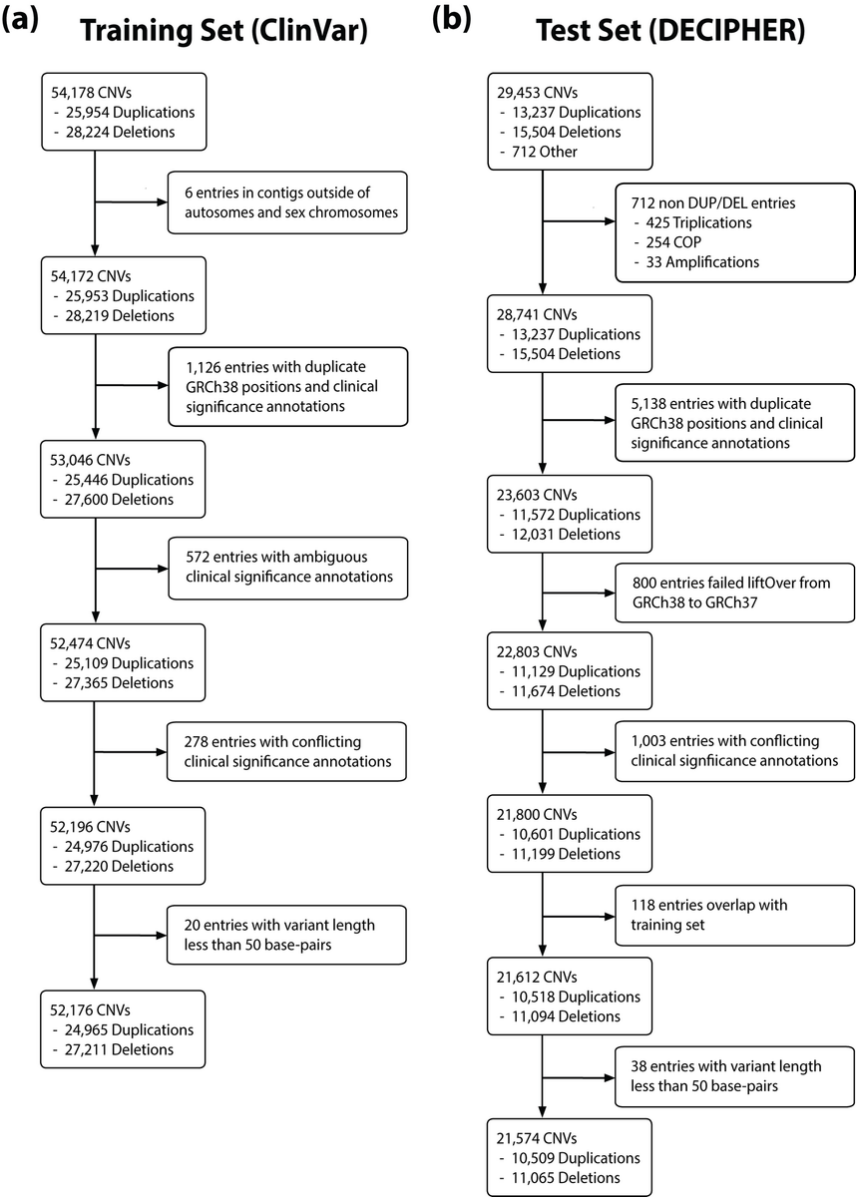
Training and Test Set Curation. CNVoyant was trained with copy number variants (CNVs) curated from ClinVar and tested on variants curated from DECIPHER. The flowcharts indicate the reasoning for omitting 2,002 variants from the training set **(a)** and 7,809 variants from the test set **(b)**. For ClinVar, 6 CNVs were mapped to contigs other than autosomes or sex chromosomes, 1,126 had matching genomic coordinates and clinical significance, 572 had ambiguous clinical significance labels, 278 variants had matching genomic coordinates and conflicting clinical significance labels, and 20 spanned less than 50 base pairs. For DECIPHER, 712 CNVs had variant types other than “duplication” or “deletion”, 5,138 had matching genomic coordinates and clinical significance, 1,003 had matching genomic coordinates and conflicting clinical significance labels, 118 overlapped with values in the training set, and 38 spanned less than 50 base pairs.

CNVoyant is trained on CNVs included in the January 2023 XML release of ClinVar (Landrum et al. 2018). This XML file was parsed, and extracted variants were limited to CNVs (variant type of “copy number gain” or “copy number loss”) that did not have duplicated variant positions. The Reference ClinVar Accession Number (RCV) entry was chosen to represent each CNV to avoid training on duplicates that can arise in cases of multiple submitters. 40,837 of the extracted CNVs were aligned to the GRCh37 reference genome, all of which required a genomic coordinate mapping via the UCSC liftOver command line tool (Hinrichs 2006) to be combined with the 12,641 CNVs that were aligned to GRCh38. Following liftOver, 1,126 variant entries were identified as duplicates of entries originally aligned to GRCh38, and they were omitted. 850 CNVs were omitted due to ambiguous clinical significance labeling or conflicting clinical significance annotation in entries with matching genomic coordinates. 20 variants were removed for having a size of less than 50 bp. The remaining ClinVar CNVs with at least one pathogenic or likely pathogenic designation were labeled as pathogenic for training purposes. Non-pathogenic variants with at least one VUS designation were labeled as VUS, and remaining variants containing only benign or likely benign classifications were labeled as benign. Altogether, 52,176 CNVs were included in model training (Fig. 2 (a), Table 1; DEL: 6,886 pathogenic, 7,191 VUS, 13,134 benign; DUP: 3,028 pathogenic, 10,792 VUS, 11,145 benign).

### Testing Dataset Curation

A test set of CNVs was curated from the web interface of the v11.18 release of the DECIPHER database (Firth et al. 2009). All 29,453 DECIPHER CNVs are aligned to the GRCh38 reference genome and have been assigned clinical significance following manual review. 712 variants had variant types other than CNV deletions or duplications and as such were removed from the test set. The remaining 28,132 variants were lifted to GRCh37 to accommodate the expected input for comparator algorithms; 800 failed to map to an autosomal or sex chromosome contig and were omitted. 1,003 entries were removed for having shared genomic coordinates and conflicting clinical significance annotation, while 5,138 entries were removed for having duplicated genomic coordinates and clinical significance annotation. To guard against data leakage between train and test sets, 118 variants whose coordinates matched a ClinVar submission were omitted from the test set. 38 variants were removed for having a size of less than 50 bp. 21,574 CNVs were included in the final test set, which was used to benchmark CNVoyant against comparator algorithms (Fig. 2 (b); DEL: 6,183 pathogenic, 3,785 VUS, 1,097 benign; DUP: 3,360 pathogenic, 5,595 VUS, 1,554 benign).

### CNVoyant Feature Selection

CNVoyant implements 17 features to classify the clinical significance of a candidate CNV. Several feature distributions are right-skewed in the training data (counts of overlapping genes, exons, promoter regions, diseases, pathogenic ClinVar SNVs/indels, and bp length). These features are log-transformed to reflect more normal distributions. All features are normalized via the sklearn preprocessing.MinMaxScaler Python class prior to training or prediction (Pedregosa et al. 2011).

#### Genomic position (2 features)

- **Centromere distance:** The number of bp separating the centromere from the candidate CNV. Distance from the centromere to the CNV is determined by selecting the CNV boundary closest to the centromere: the end coordinate on the P arm or the start coordinate on the Q arm.
- **Telomere distance**: The number of bp separating the telomere from the candidate CNV. Distance from the telomere to the CNV is calculated by selecting the CNV boundary furthest from the centromere: the start coordinate on the P arm or the end coordinate on the Q arm.

#### CNV composition (2 features)

- **GC Content**: Percentage of nucleotides in the genomic region encompassed by the candidate CNV that are guanine or cytosine.
- **BP Length**: Total bp spanning the candidate CNV.

#### Functional annotation (5 features)

- **Count of genes:** The gene count is defined as the total number of genes that overlap the candidate CNV. Genes overlap the candidate CNV if at least one bp is shared between the candidate CNV’s location and the gene’s genomic coordinates drawn from the RefSeq database (O’Leary et al. 2016). All overlap calculations were made via the Bedtools intersect function (Quinlan and Hall 2010).
- **Count of diseases**: The disease count is defined as the total number of diseases associated with the gene (s) that overlap the candidate CNV. This allows genes with more disease associations to be distinguished from genes with only a single or no disease association. Disease-gene associations are referenced from the curated annotations provided by Online Mendelian Inheritance in Man (**OMM**) (Amberger et al. 2015).
- **Count of exons**: Overlapping exon count is calculated by summing exons, across all genes, that overlap the candidate CNV. The exonic boundaries were padded by 10 base pairs to account for canonical splice regions.
- **Count of promoter regions**: CNVs may overlap the promoter region of a gene rather than the gene itself. To address this, we include a count of promoter regions, defined as the interval between the transcription start site (**TSS**) and 1,000 base pairs upstream of the TSS.
- **Count of ClinVar pathogenic SNVs and indels**: A sum of overlapping pathogenic SNV/indels is included to capture potentially relevant pathogenicity interpretation. To obtain a set of overlapping pathogenic SNV/indels, ClinVar (Landrum et al. 2014) is intersected with the candidate CNV and limited to variants interpreted as having “Pathogenic” or “Likely Pathogenic” significance.

#### Population frequency (1 feature)

- **GnomAD SV popmax**: To estimate population frequency, we identify the highest frequency across all gnomAD SV (V4) (Gudmundsson et al. 2022) entries that match the CNV’s variant type (deletion or duplication) and exhibit at least 50% reciprocal overlap in genomic coordinates. This means that the candidate CNV and the gnomAD SV entry it overlaps with must share at least half of their span, ensuring a significant genomic coverage overlap between the observed CNV and those described in gnomAD SV.

#### Conservation (2 features)

•**PhyloP**: To estimate the conservation of a candidate CNV, PhyloP scores are referenced. PhyloP scores are available at single nucleotide resolution. Single nucleotide scores are highly variable and are thus correlated with the size of the candidate CNV. To mitigate this correlation, we employ a centered moving average that considers all scores within a specified reading frame (**Supplemental Fig. 1**). This effectively smooths the otherwise volatile conservation score curve. The maximum value of this smoothed curve within the genomic coordinates of the candidate CNV is returned as the PhyloP feature. A maximum was chosen considering that higher PhyloP scores indicate higher estimated conservation.
- **phastCons**: The same procedure used to calculate the PhyloP feature was used to estimate conservation according to the phastCons score. Similar to PhyloP, the maximum was chosen as the aggregate function considering that higher phastCons scores indicate higher estimated conservation.

#### Dosage sensitivity (5 features)

- **HI Score**: Dosage sensitivity was estimated by overlapping the candidate CNV with a curated set of dosage sensitive regions described by ClinGen (Rehm et al. 2015). Manually curated HI scores are available for all curated regions. HI scores were one-hot encoded to handle each unique value and split into binary features. In the case of multiple overlapping regions, HI score features were summed.
- **TS Score**: As with HI Score, one-hot encoded binary features were generated from the curated TS scores of annotated dosage sensitive regions. The sum is also taken across all binary features when multiple dosage-sensitive regions overlap with the candidate CNV.
- **HI Index**: The minimum HI Index score (Huang et al. 2010) observed in overlapping dosage-sensitive regions is obtained to represent the HI Index value for the candidate CNV. HI Index estimates the probability of loss intolerance, where lower values predict haploinsufficiency.
- **pLI**: The maximum pLI score (Exome Aggregation Consortium et al. 2016) observed in overlapping dosage-sensitive regions is obtained from gnomAD to represent the pLI value for the candidate CNV. pLI estimates the probability of loss intolerance, where higher values predict haploinsufficiency.
- **LOEUF**: The minimum LOEUF score (Karczewski et al. 2020) observed in overlapping dosage-sensitive regions is obtained to represent the LOEUF value for the candidate CNV. LOEUF estimates the probability of loss intolerance, where lower values predict haploinsufficiency.

### Training Procedure and Model Selection

For model training, features were generated for CNVs included in the ClinVar training set before partitioning into deletion and duplication sets. Separate multi-class models were trained for duplication and deletions, each predicting whether a candidate CNV has benign, uncertain, or pathogenic clinical significance. 29 common ML architectures were tested via 5-fold cross-validation for each CNV type before selecting the top performers according to multi-class F1 score. Following architecture selection, 5-fold cross-validation was again employed to hyperparameter tune and find the most accurate model. 10,000 sets of hyperparameter values were tested for each CNV type via the sklearn.model_selection.RandomizedSearchCV class. The top-performing models were calibrated to class distributions in the training set via the isotonic method implemented in the sklearn.calibration.CalibratedClassifierCV class.

### Comparator Algorithm Selection

CNVoyant predictions were compared to five published ML-based CNV pathogenicity classifiers, X-CNV (Zhang et al. 2021), TADA (Hertzberg et al. 2022), dbCNV (Lv et al. 2023), StrVCTVRE (Sharo et al. 2022), and ISV (Gažiová et al. 2022), as well as the algorithmic implementation of the ACMG technical standards for CNV interpretation, ClassifyCNV (Gurbich and Ilinsky 2020). The test set was passed to CNVoyant and all comparator algorithms to test for generalizability and generate accuracy metrics for benchmarking. Given that X-CNV, TADA, dbCNV, and ClassifyCNV take GRCh37-aligned variants as input, UCSC liftOver was again called to lift DECIPHER variants from GRCh38 to GRCh37. X-CNV pathogenic probabilities yielded only three unique values across the entirety of the test set, which was unexpected given the generation of a relatively heterogeneous feature set. Rather than attempting to amend the source code to produce more continuous output, X-CNV was omitted from benchmarking.

### Measuring Performance

To generate granular performance data in the test cohort, accuracy metrics are reported for each of the three CNV prediction classes: benign, VUS, and pathogenic. The classification probability for each class was utilized to sort the list of CNVs and generate precision-recall (**PR**) and receiver operating characteristic (**ROC**) curves. The area under these curves (**PR AUC, ROC AUC**) is referenced to measure model accuracy, in addition to the average F1 score and overall accuracy for multi-class predictions. F1 and overall accuracy are only reported for CNVoyant, dbCNV, and ClassifyCNV, as these algorithms are the only three that provide multi-class output. dbCNV provides likely pathogenic and likely benign classification designations in addition to pathogenic, VUS, and benign designations. Likely pathogenic predictions were mapped to pathogenic classification and likely benign predictions were mapped to benign classification to ensure a fair comparison to CNVoyant and ClassifyCNV.

TADA, ISV, and StrVCTVRE all output a single score to estimate CNV pathogenic probability. The complement of the pathogenic probability score (1-Pr (pathogenic)) was calculated to estimate benign significance scores for these comparator algorithms. The ClassifyCNV output is a score rather than a probability, but the complement was still chosen to represent benign significance, as higher scores represent a higher pathogenicity. CNVoyant is the only algorithm that provides benign and VUS probabilities; these values were used in plotting corresponding benign and VUS classification curves. dbCNV does not provide probabilities or a continuous confidence score for classification, so there is no value to reference in plotting ROC and PR curves. As such, dbCNV was not included in the ROC and PR curve comparisons.

### Deciphering Feature Influence on CNV Classification with SHAP Analysis

SHAP (**SH**apley **A**dditive ex**P**lanations) values offer a qualitative analysis tool for understanding how each feature influences the clinical significance prediction for distinct CNV classes. For CNVoyant, we generated SHAP beeswarm plots across all classes to visualize the effect of training features on model prediction (Lundberg and Lee 2017). These plots rank features by their importance and use color coding to depict the direction of their influence on the model’s output. Each point on a plot represents a feature’s SHAP value for an individual observation, quantifying its contribution to moving the model’s prediction from the base value—the dataset’s average prediction—toward the actual prediction.

## Results

**Figure 3:**
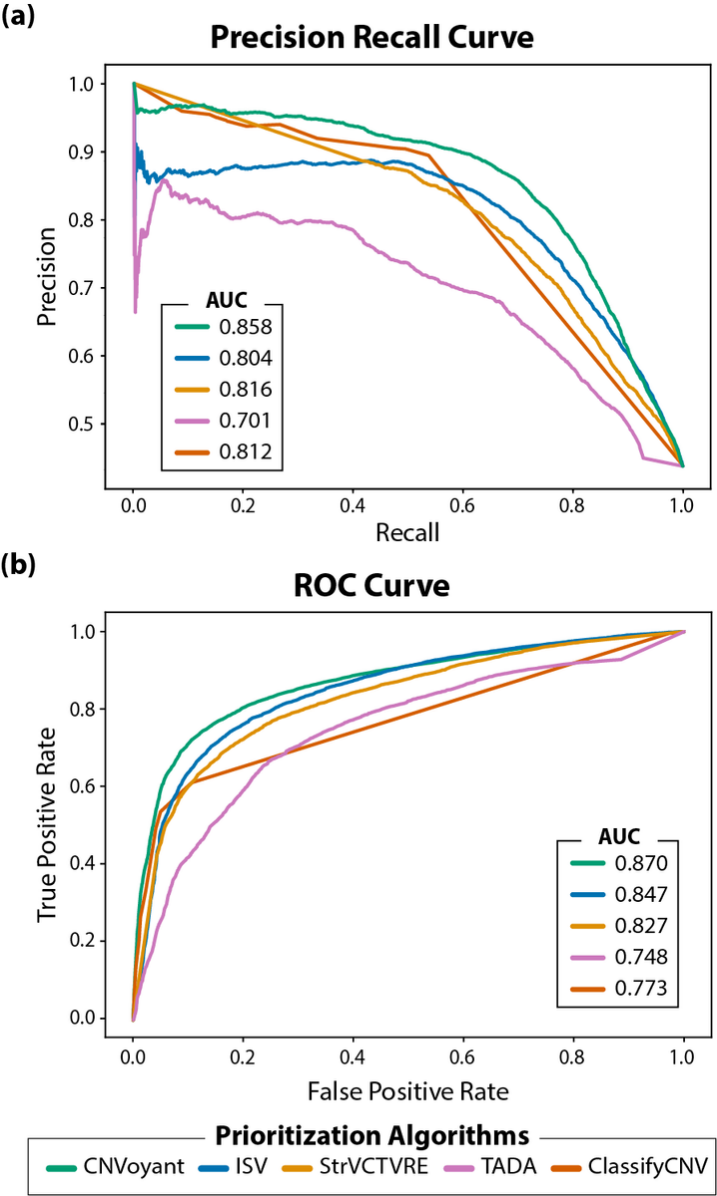
Binary Classification of Pathogenic Copy Number Variants. The performance of CNVoyant was compared to four algorithms (ISV, StrVCTVRE, TADA, ClassifyCNV) in the binary classification of pathogenic CNVs. The discriminative power of each algorithm is quantified using the area under the curve (AUC) from both **(a)** precision-recall (PR AUC) and **(b)** receiver operating characteristic (ROC AUC) curves. CNVoyant demonstrates superior performance in distinguishing pathogenic from non-pathogenic CNVs, achieving the highest PR AUC of 0.858, indicating its effectiveness in correctly identifying pathogenic CNVs with a high degree of precision and recall. The rankings for PR AUC performance are as follows: CNVoyant (0.858), StrVCTVRE (0.816), ClassifyCNV (0.812), ISV (0.804), and TADA (0.701). Similarly, CNVoyant leads in ROC AUC with a score of 0.870, showcasing its overall capability to accurately classify CNVs across different thresholds. The ROC AUC rankings are: CNVoyant (0.870), ISV (0.847), StrVCTVRE (0.827), ClassifyCNV (0.773), and TADA (0.748).

Our evaluation of multiple machine learning architectures culminated in selecting a random forest model for both duplication and deletion CNV predictions, setting the stage for a comprehensive analysis of CNVoyant’s performance against other leading algorithms. The performance of each algorithm was evaluated using PR AUC and ROC AUC metrics, highlighting the effectiveness of CNVoyant in distinguishing between benign, VUS, and pathogenic CNVs. For pathogenic CNV entries in the test set, CNVoyant displayed the highest PR AUC (0.858) and ROC AUC (0.870) (Fig. 3 (a-b), Table 2). StrVCTVRE displayed the second-highest PR AUC (0.816), followed by ClassifyCNV (0.812), ISV (0.804), and TADA (0.701). In terms of ROC AUC, ISV was the second-most accurate algorithm (0.847), followed by StrVCTVRE (0.827), ClassifyCNV (0.773), and TADA (0.748). For benign CNV entries in the test set, CNVoyant displayed the highest PR AUC (0.463), followed by StrVCTVRE (0.461), ClassifyCNV (0.373), ISV (0.344), and TADA (0.282) (**Supplemental Fig. 2 (a-b)**). In terms of ROC AUC, CNVoyant was again the most accurate algorithm (0.848), followed by ISV (0.819), StrVCTVRE (0.817), TADA (0.751), and ClassifyCNV (0.689). CNVoyant’s accuracy in classifying VUS was respectable but less accurate than pathogenic classification (PR AUC: 0.642; ROC AUC: 0.757). Similarly, CNVoyant benign classification was also less accurate than pathogenic classification.

**Figure 4:**
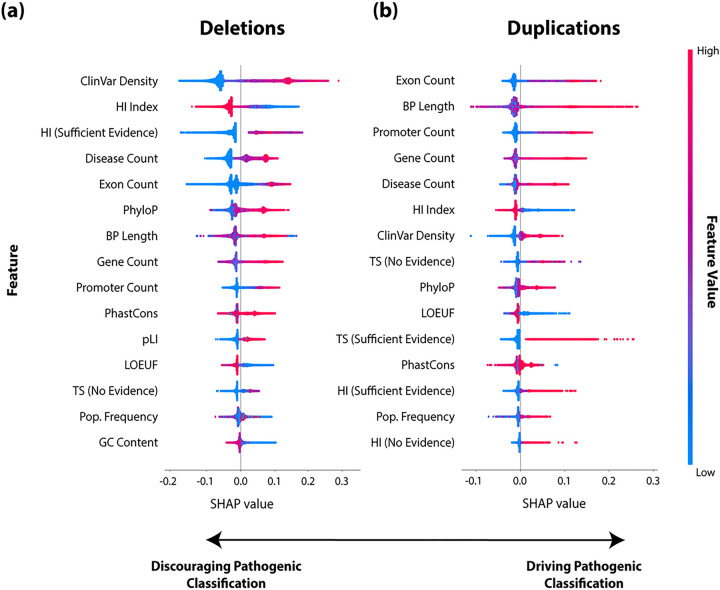
SHAP Beeswarm Plots for CNVoyant Pathogenic Classification. SHapley Additive exPlanations (SHAP) values are provided to illustrate the impact of genomic features on the machine learning classification of CNVs SHAP values offer a measure of each feature’s contribution to the model’s prediction, with higher absolute values indicating greater influence. Separate models were trained for **(a)** CNV deletions and **(b)** duplications; beeswarm plots are provided for each. Each point in the graph indicates a feature value for a specific training CNV. Positive SHAP values indicate that features support a pathogenic classification, and negative values detract from a pathogenic classification. The color intensity reflects the magnitude of feature values. Features are displayed in descending order by influence on the model’s decision. Detailed feature descriptions are provided in the CNVoyant Feature Selection section of the Methods.

The most informative features in the SHAP beeswarm plots for pathogenic classification differed between deletion and duplication events (Fig. 4). Pathogenic SNV/indel overlap was the most important feature for deletions, and exon count was the most important feature for duplications. For deletions, the HI index and a curated HI score of “sufficient evidence” were the second and third most important features, respectively. For duplications, bp length and promoter region count were the second and third most important features, respectively. The top five most informative features between deletions and duplications included exon count and disease count. PhyloP was more informative than phastCons in both variant types but was more important in predicting pathogenic deletions (6th most important feature) than pathogenic duplications (9th most important feature). SHAP beeswarm plots for benign and VUS classification indicated more similar feature importance between duplication and deletion variants (**Supplemental Fig. 3**, benign (a-b) and VUS (c-d)). For benign classification, the top four features were shared between duplication and deletion variants in the same order of importance. Bp length was the most important feature, followed by pathogenic SNV/indel overlap, PhyloP, and exon count. For VUS classification, bp length was the most important feature, followed by exon count for both duplications and deletion variants. PhyloP was the third most important feature for deletion events, followed by gene count. Pathogenic SNV/indel overlap was the third most important feature for VUS classification in duplications, followed by PhyloP. Population frequency and GC content were relatively uninformative across benign, VUS, and pathogenic predictions.

**Figure 5:**
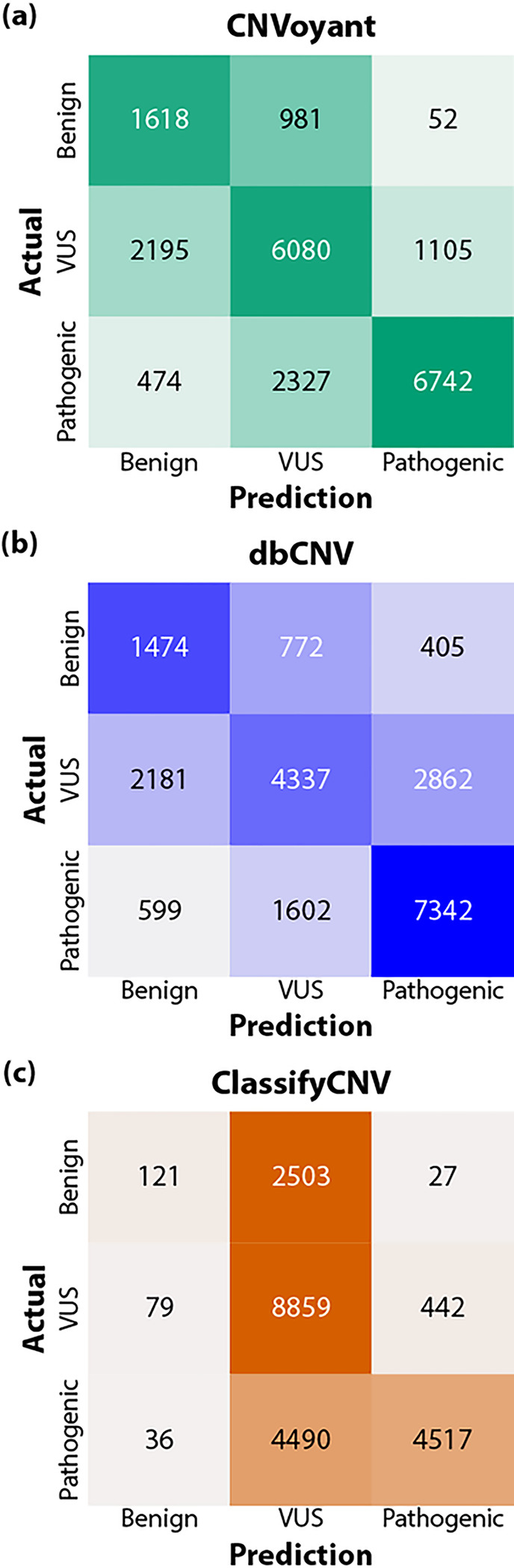
Multi-Class Confusion Matrices for CNV Classification. This visualization presents confusion matrices for CNVoyant, dbCNV, and ClassifyCNV, showcasing the algorithms’ ability to classify CNVs into multiple categories. The matrices illustrate the correlation between actual categories (row-wise) and predicted categories (column-wise), with color intensity indicating the proportion of observations normalized by the totals for actual labels. Darker shades denote higher proportions, highlighting the model’s classification capability per category. Ideally, a perfect classifier would have all observations along the diagonal line from the top left to the bottom right, indicating accurate category prediction for every observation. Among the algorithms capable of multi-class predictions, CNVoyant outperforms the others, demonstrating more precise classification across different CNV categories. Specifically, CNVoyant exhibits the most effective classification of benign and pathogenic CNVs, with F1 scores of 0.466 and 0.773, respectively. This compares favorably to dbCNV, with benign and pathogenic F1 scores of 0.427 and 0.729, and ClassifyCNV, with significantly lower scores of 0.084 for benign and 0.622 for pathogenic CNVs. Notably, while ClassifyCNV shows a preference for variants of uncertain significance (VUS) predictions with an F1 score of 0.689, it underperforms in benign CNV classification. CNVoyant not only leads in category-specific F1 scores but also achieves the highest overall accuracy rate of 0.669, indicating a greater proportion of correct predictions across all categories, compared to ClassifyCNV (0.626) and dbCNV (0.610). Additionally, CNVoyant maintains the highest average F1 score across categories (0.629), evidencing its superior balanced performance across benign, pathogenic, and VUS classifications, in contrast to dbCNV (0.565) and ClassifyCNV (0.465), which exhibit lower average F1 scores.

In our analysis, CNVoyant demonstrated superior accuracy in multi-class clinical significance classification (overall accuracy: 0.669, average F1: 0.629) compared to dbCNV (overall accuracy: 0.610, average F1: 0.565) and ClassifyCNV (overall accuracy: 0.626, average F1: 0.465) (Fig. 5). When only considering benign variants, CNVoyant (F1: 0.466) outperformed dbCNV (F1: 0.427) and ClassifyCNV (F1: 0.084). ClassifyCNV was the most accurate model in predicting VUS CNVs (F1: 0.689), followed by CNVoyant (F1: 0.648) and dbCNV (F1: 0.539). For pathogenic CNVs, CNVoyant was the most accurate (F1: 0.773), followed by dbCNV (F1: 0.729) and ClassifyCNV (F1: 0.622).

## Discussion

CNVoyant sets a new standard in the classification of clinical significance for CNVs. Our novel algorithm outperformed the five leading algorithms for classifying CNVs (ClassifyCNV, ISV, StrVCTVRE, TADA, dbCNV) across all accuracy metrics and clinical significance classes in the DECIPHER test set. This unparalleled accuracy underscores CNVoyant’s advanced analytical capabilities, especially when considering the complexity and variability resulting from individual provider submissions within the DECIPHER data set. Furthermore, for the first time, our comprehensive evaluation of feature performance within the predictive model has uncovered novel insights into the determinants of CNV clinical significance, offering a deeper understanding of the underlying drivers of classification.

CNVoyant prediction probabilities closely align with the observed class distributions in the DECIPHER test set (**Supplemental Fig. 4**), supporting the generalizability of these predictions. Regarding explainability, the SHAP values generated from the test set also reflect intuitive reasoning driving predictions. Larger CNVs overlap with more functional and dosage sensitive regions, which are logically more likely to be pathogenic, and this was clearly reflected in the pathogenic SHAP beeswarm plots (Fig. 4 and **Supplemental Fig. 3**). Conversely, an inverse relationship exists where smaller CNVs that overlap with fewer regions drive benign predictions. The length of a candidate CNV was a simple but highly important feature omitted from ISV, StrVCTVRE, and TADA. Specifically, bp length was the most informative feature for both deletions and duplications in both VUS and benign variants. We hypothesize that a portion of the overall performance gained over the comparator algorithms was due to the addition of this feature.

In deletions, the count of pathogenic SNVs and indels contained within the CNV boundaries was the most important feature in predicting pathogenic significance and the second most important feature in predicting benign significance. This is also to be expected, as regions more intolerant to variation have more disease-causing variants. Given that loss of function is the most common variant type of pathogenic or likely pathogenic ClinVar SNV and indel annotations (72.5% of such variants), the emphasis placed on deletion events aligns with expectations. This trend was further observed in the context of conservation, with deletion variants spanning highly conserved regions having more pathogenic potential. Interestingly, HI and conservation metrics showed predictive value in classifying duplication variants. After further investigation, it was recently reported that HI and TS features largely overlap, confirming our observed trend (Collins et al. 2022).

As previously stated, ClassifyCNV is an algorithm that encodes the logic driven by the most recent ACMG technical standards for CNV interpretation. While there is value in minimizing false positive predictions, especially in clinical settings, the consequence is less accuracy in identifying true negatives. We observed this lack of true negative recall in the DECIPHER cohort, where ClassifyCNV predicted that only 4.7% of the 2,651 benign CNVs had benign clinical significance. This effectively leaves 98.9% of called CNVs to be interpreted by clinical genomicists, a value that does not significantly reduce the burden of variant interpretation. ML methods can address this issue, as they can consider features that are not included in the current clinical guidelines. Comparator models each have certain blind spots that CNVoyant aims to account for. TADA fails to contextualize the genomic position of the CNV itself and instead focuses on overlapping topologically associating domains. ISV and StrVCTVRE address these shortcomings but fail to consider reported pathogenic SNVs and indels in ClinVar. Echoing the principle of Occam’s Razor, CNVoyant underscores the power of simplicity by leveraging a concise set of features to outperform more complex models. This approach streamlines the analytical process and enhances the model’s explainability, affirming the notion that simplicity often leads to superior outcomes.

The potential for variability in the rigor with which clinical significance is assigned within the DECIPHER dataset reflects one potential shortcoming of our study. CNVs in this test set were submitted by individual providers, and submitters likely used varying methods to assess the clinical significance of a given CNV. Given the vast size of this test dataset (21,574 CNVs) and the challenges of reassessing all these CNVs with a standardized set of guidelines, we had to accept the assigned significance label in our test data.

In the realm of clinical genomics, experts frequently encounter CNVs that may straddle the line between different clinical significance classes. In these cases, understanding the rationale behind ML classification can be invaluable. CNVoyant addresses this need by providing a novel feature amongst existing CNV classification algorithms, SHAP force plots (**Supplemental Fig. 5**). This approach enables CNVoyant to effectively highlight the CNV features with the greatest influence on the model’s classification decision, providing critical insights to guide clinical interpretation. With this in mind, we engineered CNVoyant to export the plots into portable static image files, which can easily be attached to clinical notes or reports.

It should be noted that CNVoyant alone cannot predict the diagnostic significance of a patient’s CNVs. Other factors must be considered, including variant zygosity, phenotypic overlap with associated diseases, mode of inheritance of associated diseases, presence of additional variants in trans, and differences in CNV frequencies in clinical settings enriched with patients affected by genetic conditions. CNVoyant should instead be included in diagnostic classification architectures to limit candidate diagnostic CNVs to only those with requisite probabilities of pathogenicity. CNVoyant outputs probabilities of benign, uncertain, and pathogenic significance, which are predicated on label distributions in the training set. In clinical cases, there are far more benign variants than variants with uncertain or pathogenic significance. Often, a patient will only have CNVs of benign clinical significance. To combat this class imbalance, CNVoyant should be retrained on interpreted CNVs from real patients before implementation in a clinical decision-support setting. While the current catalog of annotated CNVs is limited, it will undoubtedly grow exponentially as more CNVs are detected and interpreted in clinical settings. In anticipation of new data, we have open-sourced CNVoyant’s source code to allow users to train models with new data using the same feature set and architecture.

Finally, it should be reiterated that CNVs are only one category within the larger domain of structural variation. Additional pathogenicity prediction algorithms are required to predict the clinical significance of other types of structural variation, including inversion and translocation events. Comprehensive variant prioritization algorithms must account for all structural variant types and simultaneously consider shorter variants, including SNVs and indels.

## Conclusions

The advent of GS technologies and advanced algorithms has revolutionized our ability to detect genetic variants, including segmental duplications and deletions, reliably. Clinical genomics experts must painstakingly interpret these CNVs to determine their relevance to a patient’s suspected genetic condition. To aid this process, we introduce CNVoyant, a highly accurate algorithm for classifying the clinical significance of CNVs. CNVoyant’s unparalleled accuracy in classifying CNVs’ clinical significance is driven by a unique ML architecture and a carefully selected set of features that capture the multitude of factors that should be considered when evaluating the impact of a CNV. Importantly, CNVoyant demystifies ML decisions through SHAP force plots, providing the rationale behind the algorithm’s classification for any given CNV and enhancing transparency for clinicians. With the source code publicly available, CNVoyant invites continuous evolution, allowing for retraining with new data or specific populations. This adaptability will ensure that CNVoyant remains at the forefront of genomic medicine, simplifying variant prioritization and scaling to meet the demands of expanding GS applications. CNVoyant not only sets a new standard for accuracy and explainability, but also advances the capability to discern pathogenic significance, marking a significant leap in genomics.

## Figures and Tables

**Table 1:** Distribution of Variant Type and Clinical Significance in Training and Test Sets. 52,176 total CNVs were included in the ClinVar training set, and 21,574 total CNVs were included in the DECIPHER test set. The training set generally favored variants of benign significance, with pathogenic significance encompassing the fewest number of variants. This trend was reversed in the test set, which heavily favored VUS and pathogenic CNVs over those with benign significance. Clinical significance class distribution was generally consistent between duplication and deletion events except for more pathogenic variants being present in deletions.

		Benign	VUS	Pathogenic	Total
Deletions	**Train**	13,134 (48.3%)	7,191 (26.4%)	6,886 (25.3%)	27,211
	**Test**	1,097 (9.9%)	3,785 (34.2%)	6,183 (55.9%)	11,065
Duplications	**Train**	11,145 (44.6%)	10,792 (43.2%)	3,028 (12.2%)	24,965
	**Test**	1,554 (14.8%)	5,595 (53.2%)	3,360 (32.0%)	10,509
Total	**Train**	24,279 (46.5%)	17,983 (34.5%)	9,914 (19.0%)	52,176
	**Test**	2,651 (12.3%)	9,380 (43.5%)	9,543 (44.2%)	21,574

**Table 2:** Benchmarking Algorithmic Classification of CNV Clinical Significance. The classification score performance of CNVoyant was compared to four algorithms (ISV, StrVCTVRE, TADA, ClassifyCNV) in determining the clinical significance of deletions, duplications and combined CNVs. The effectiveness of each algorithm was assessed by calculating the area under the curve (AUC) for both the precision-recall (PR AUC) and the receiver operating characteristic (ROC AUC). These metrics were selected to provide a comprehensive evaluation of each classifier’s ability to discriminate between clinically significant and non-significant CNVs under various threshold settings. CNVoyant demonstrated superior performance to all compared algorithms across most CNV subsets as evaluated by these metrics, except for StrVCTVRE, which exhibited a higher PR AUC in classifying benign deletion variants. dbCNV was excluded from this comparison due to the absence of a continuous variable necessary for plotting PR and ROC curves.

		Benign		VUS		Pathogenic	
		PR AUC	ROC AUC	PR AUC	ROC AUC	PR AUC	ROC AUC
Deletions	**CNVoyant**	0.4735	**0.8904**	**0.5971**	**0.7827**	**0.9028**	**0.8850**
	**ISV**	0.4060	0.8719	---	---	0.8456	0.8481
	**StrVCTVRE**	**0.5124**	0.8663	---	---	0.8738	0.8422
	**TADA**	0.2908	0.8179	---	---	0.7750	0.7634
	**ClassifyCNV**	0.3799	0.7641	---	---	0.8675	0.8077
Duplications	**CNVoyant**	**0.4592**	**0.8002**	**0.6650**	**0.6939**	**0.7512**	**0.8216**
	**ISV**	0.3328	0.7694	---	---	0.7086	0.8117
	**StrVCTVRE**	0.4249	0.7603	---	---	0.7086	0.7847
	**TADA**	0.2784	0.6720	---	---	0.4500	0.6699
	**ClassifyCNV**	0.3674	0.6021	---	---	0.7012	0.6788
Combined	**CNVoyant**	**0.4630**	**0.8478**	**0.6415**	**0.7573**	**0.8582**	**0.8705**
	**ISV**	0.3441	0.8192	---	---	0.8039	0.8474
	**StrVCTVRE**	0.4608	0.8165	---	---	0.8155	0.8268
	**TADA**	0.2824	0.7506	---	---	0.7012	0.7483
	**ClassifyCNV**	0.3727	0.6889	---	---	0.8119	0.7725

## Data Availability

ClinVar XML files used to generate the training set are available via the provided FTP site (https://ftp.ncbi.nlm.nih.gov/pub/clinvar/xml/), and DECIPHER variants are available via the web-based graphical user interface (https://www.deciphergenomics.org/). CNVoyant is published under an Open Source Initiative approved 3-Clause BSD License to ensure that any interested academic institution can perform optimization with their cohorts and implement their version of the algorithm in their respective diagnostic workflows. The code for the CNVoyant algorithm is available in our GitHub repository (https://github.com/nch-igm/CNVoyant). It is also available via the pip package manager (https://pypi.org/project/CNVoyant/) and conda package manager in Linux and OS distributions (https://anaconda.org/schuetz.12/cnvoyant).

## Abbreviations

ACMG: American College of Medical Genetics
AUC: Area under the curve
CNV: Copy number variant
ES: Exome sequencing
GS: Genomic sequencing
HI: Haploinsufficiency
Indel: short insertion or deletion variant
MI: Machine learning
PR: Precision-recall
ROC: Receiver operating characteristic
SHAP: SHapley Additive exPlanations
SV: Structural variant
SNV: Single nucleotide variant
TS: Triplosensitivity
VUS: Variant of uncertain significance
